# Study on the water-richness law and zoning assessment of mine water-bearing aquifers based on sedimentary characteristics

**DOI:** 10.1038/s41598-022-18403-5

**Published:** 2022-08-18

**Authors:** Yang Wang, Zhiguo Pu, Qin Ge, Jinhui Liu

**Affiliations:** 1 State Key Laboratory of Nuclear Resources and Environment, East China University of Technology, Nanchang, 330013 China; 2 School of Water Resources & Environmental Engineering, East China University of Technology, Nanchang, 330013 China; 3 China Coal Energy Research Institute Co., Ltd., Xi’an, 710054 China; 4 China Coal Rock Burst & Water Hazard Control Center, Ordos, 017000 China

**Keywords:** Hydrology, Natural hazards, Solid Earth sciences, Energy science and technology

## Abstract

To study and prevent the water hazards of deep coal mines roof in the Inner Mongolia–Shaanxi (IM–S) mining area, it is essential to correctly evaluate the water-richness distributions of water-bearing aquifers in roof. This paper puts forward a sediment control method for water-richness law and zoning in the roof aquifers of deep Jurassic coals. To determine the vertical distance of direct water-bearing aquifers, the height of fractured water-conducting zone was detected by an underground network parallel electrical method. The plane and lateral spatial distribution patterns of the water-bearing aquifers and the control of the water-richness distribution was analyzed with the sediment control method. An evaluation system that consisted of four indicators, i.e., sedimentary environmental impact index, interlayer ratio of sandstone and mudstone, sandstone thickness, geophysical water-richness anomaly index was constructed. Furthermore, an Analytic Hierarchy Process (AHP) was introduced to establish the comprehensive zoning map. Finally, through the example analysis of Muduchaideng coal mine, the zoning evaluation results of water-richness were verified by the mine inflow. The findings of this study provide scientific guidance for prevention and control of mine water hazards in the IM–S mining area.

## Introduction

Geological reserves of Jurassic coals account for about 60% of the total coal resources in China, and are mainly distributed in the northwest regions. Among them, the estimated reserve in the Ordos Basin is the largest in China^1–3^. Specifically, the Yulin-Hengshan mine, the Nalinhe mine and the Huji’erte mine are located within the boundary of the Mongolia–Shaanxi (IM–S) mining area and command huge coal reserves in excellent quality. They are newly-developed areas of deep coal seams, the majority of which are generally buried as deep as 600–800 m^4,5^. Water-richness of the water-bearing aquifers is unevenly distributed in the deep mines of the IM–S mining area because the Jurassic strata underwent multi-phase transformations and progressive sedimentation processes, forming a feature of a strip shape on the plane with lateral staggered deposition, which has resulted in complex hydrogeological conditions in the mines and exposure of the mines to the severe threat posed by roof water-related hazards^6–8^. Therefore, it is of great practical significance to make a reasonable evaluation and prediction of water-richness distribution of aquifers for safe mining in deep mines.

Scholars have carried out many studies on roof water-related hazards in this area. These studies mainly focused on the sedimentation characteristics of the Jurassic strata and water-richness evaluation of water-bearing aquifers^9^. Xu et al. systematically investigated the characteristics of water-bearing media in Jurassic aquifers, including sedimentary environment, microstratigraphic structure, and mesoscopic pore structure^10^. Wang et al. investigated the relationship between the microscopic pore structure and the water-richness of Jurassic sandstone in the Ningtiaota coal mine of the Jurassic coalfield in northern Shaanxi Province by using different experimental test techniques, such as ordinary thin section, cast thin section, high-pressure mercury injection, and nuclear magnetic resonance^11^. According to different data sources, scholars have put forward different methods to zone water-richness of water-bearing aquifers. These methods can be divided into three categories, i.e., geophysical prospecting method^12,13^, pumping test method^14,15^ and multi-factor comprehensive method^16,17^. Because the geophysical prospecting method and the pumping test method have some disadvantages, such as large engineering amount, high cost, and limited control scope, the multi-factor comprehensive method is more widely used^18^. The water-richness index method proposed by Wu et al. employed a GIS-based method to combine multi-source information by identifying the main factors that affect aquifer water richness^19^. The hydrogeological information of mines can be fully exploited with water-richness index method.

Different from the Carboniferous-Permian coal-accumulating basins in eastern China, the Ordos Jurassic coal-accumulating basin in western China is a complete set of fluvial-lacustrine terrigenous clastic strata with weak internal tectonic activities. The sedimentary environment is an important factor controlling the distribution of aquifers in the Jurassic strata of Shaanxi-Inner Mongolia mining area^20–22^. Previous researches on the sedimentation characteristics of the Jurassic strata mainly focused on the evolution pattern of the microscopic pore structure of sandstone. The influence of sedimentation characteristics on the water-richness distribution in the aquifers of the mines was limitedly known. Therefore, the objectives of this study are to, (i) investigate the spatial distribution and water-richness distribution of roof water-bearing aquifers, (ii) construct and define the evaluation indicator of sedimentary environmental impact index, (iii) propose a quantitative evaluation method for determination of water-richness of roof water-bearing aquifers in deep Jurassic mines.

## Study area

**Figure 1:**
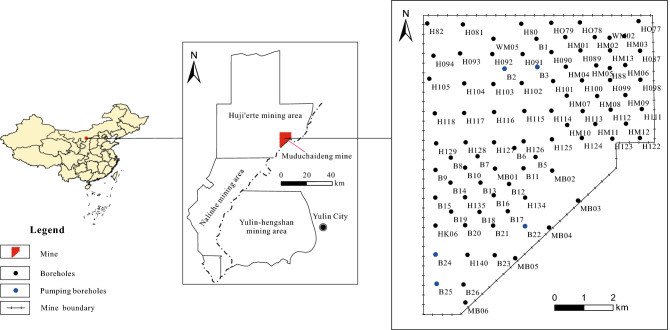
Location of the study area in the Huji'erte mining area. Maps were generated using ArcGIS 10.1 for Desktop (http://www.esri.com/sofware/arcgis/arcgis-for-desktop).

Muduchaideng Coal Mine is selected as the study area of this study. The mine is located in the Huji'erte mining area of the Dongsheng Coalfield inside the Inner Mongolia Autonomous Region of China. It belongs to Tuke Town of the Wushen Banner in Ordos City, 57 km southwest to Wushen Banner and 75 km southeast to Yulin City of Shaanxi Province (Fig. 1). The stope is seated in the southern edge of the Mu Us Desert. The transition zone is located between Inner Mongolia and Shaanxi Province with the typical features of a plateau desert landform. The ground surface is completely covered by Quaternary wind-deposited sands and is dotted by sparse vegetation and free of bedrock outcrops. The well site is shaped like an irregular polygon, covering an area of 59.6 km^2^ with the longest north–south length of about 10.2 km and the widest east–west width of about 7.9 km. The coal resources in the well site are 1116 Mt, of which the recoverable reserves are 704 Mt with a designed production capacity of 6.0 Mt/a.

**Figure 2:**
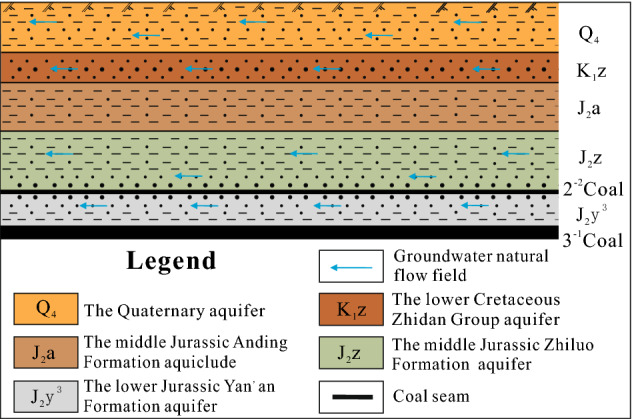
Hydrogeological section of the study area.

According to drilling data, the stratas in the well site are the middle Jurassic Yan’an Formation (J_2_y), the middle Jurassic Zhiluo Formation (J_2_z), the middle Jurassic Anding Formation (J_2_a), the lower Cretaceous Zhitan Group (K_1_z), and the Quaternary (Q_4_) from the bottom to the top, respectively (Fig. 2). The Yan’an Formation in the middle Jurassic strata are coal-bearing and contains 8 mineable coal seams. Presently, the main mining seam is the 3^–1^ seam. According to the drilling of pumping tests and regional hydrogeological data, the water-bearing media are divided into four groups: the loose unconfined Quaternary aquifer (I), the pore-fracture confined aquifer of lower Cretaceous Zhidan group (II), the pore-fracture confined aquifer of middle Jurassic Zhiluo Formation (III), and the pore-fracture confined aquifer of middle Jurassic Yan’an Formation (IV).

## Methodology

The sediment control method proposed in this paper mainly consists of two parts: the analysis of the controlling effect of sedimentary characteristics on the water-richness of roof aquifers and the evaluation of water-richness zoning based on sedimentary characteristics. Based on this framework, the proposed sedimentary water control method is divided into four steps.

The first step is to analyze the spatial distribution and water-richness distribution of water-bearing aquifers. Firstly, through the use of an underground network parallel electrical method, we can determine the vertical distance of direct water-bearing aquifers, and the sedimentary facies types of them are divided in detail by using the geological boreholes data of the study area. Then, we analyze their spatial distribution and water-richness distribution from genesis by categorizing the types and distributions of the sedimentary facies.

The second step is to construct a set of comprehensive evaluation indicator system. As the factors affecting the water-richness of roof aquifers are often multi-dimensional and complex, we can construct four indicators, i.e., sedimentary environmental impact index, interlayer ratio of sandstone and mudstone, sandstone thickness, and geophysical water-richness anomaly index, to evaluate the water-richness of roof aquifers.

The third step is to establish the water-richness zoning assessment model. To simplify the evaluation process and avoid uncertainties caused by excessive emphasis on internal changes of various indicator values, we can build a more mature and easily-operated weighting model based on AHP, the details of which have been published in Wu et al.^14^.

The fourth step is the analysis and verification of evaluation results. According to the “Rules of Coal Mine Water Prevention and Control”, the results of the water-richness zoning evaluation of roof aquifers are verified with limited unit water inflow of pumping boreholes or mine water inflow. If the evaluation results are consistent with the measured unit water inflow or mine water inflow, it means that the evaluation results are reasonable and effective. Otherwise, it is necessary to identify each information thematic map by eliminating invalid boreholes data and adjusting the interval threshold of each evaluation indicator; on the other hand, the weights of each evaluation indicator should be adjusted according to the geological and hydrogeological conditions of the study area until it is consistent with the actual evaluation grade.

The specific meanings of the four evaluation indicators in the second step are indicated as follows.

### Sedimentary environmental impact index

The indicator proposed in this paper is intended to quantitatively depict the impact of the distribution of sedimentary facies on the water-richness distribution of water-bearing aquifers. According to the pumping test results, the ratio of sandstone thickness to stratum thickness used to characterize the distribution of sedimentary facies in different aquifers is compounded and superimposed.

### Interlayer ratio of sandstone and mudstone

Under the condition that the thickness of the aquifer is constant, the more layers between sandstone and mudstone, the weaker the hydraulic connection between groundwater within the aquifer will be, which in turn leads to a decrease in the water-richness degree of the aquifer. On the contrary, if the interlayer ratio of sandstone and mudstone decreases, the thickness of the corresponding monolayer sandstone will increase, and the connected sand bodies are easily formed into the plane, so that the water-richness degree of the aquifer is enhanced.

### Sandstone thickness

Generally, when other factors are constant, the thickness of sandstone is directly proportional to the water-richness degree of the aquifer. The larger the thickness of sandstone is, the larger the space for groundwater storage per unit thickness of sandstone will be, and the water-richness degree of the aquifer will be stronger.

### Geophysical water-richness anomaly index

Geophysical exploration can accurately detect the water-richness of the aquifer, which can effectively make up for the gap between regional data other than geological and hydrogeological boreholes data and reduce the error of data interpolation in the water-richness evaluation. According to the distribution pattern of geophysical anomaly characteristics interpreted by ground transient electromagnetic method (TEM), the indicator is used to quantitatively depict the application of geophysical results in evaluating the water-richness of the aquifer by experts scoring and assigning values.

**Figure 3:**
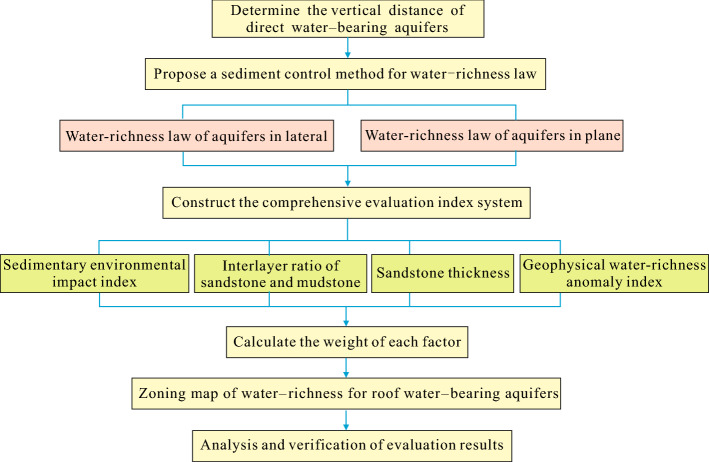
Flowchart of the methodology used in this study.

A detailed flowchart of the methodology used in this study is shown in Fig. 3.

## Results and discussion

### Controlling effect of sedimentary characteristics on the water-richness of water-bearing aquifers

#### Determination of the vertical distance of direct water-bearing aquifers

**Figure 4:**
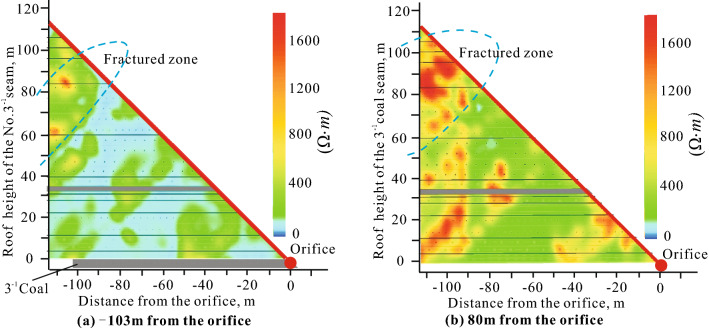
Results of drilling apparent resistivity.

To evaluate the water-richness of direct water-bearing aquifers in coal roof, it is necessary to detect the height of fractured water-conducting zone, so as to determine the vertical distance of the water-richness evaluation. To accurately and intuitively analyze the failure process and development characteristics of rock mass, we use an underground network parallel electrical method. The development height of fractured zone ranges from 50 to 90 m when the 3^–1^ coal seam in the first mining face is mined to -103 m from the orifice (Fig. 4a). When the 3^–1^ coal seam is pushed past the borehole for a distance, the development height of fractured zone is gradually stabilized. When the height of fractured zone ranges from 50 to 106 m, and the apparent resistivity in this range generally rises to more than 600 ohms, which is a typical resistivity characteristic of the fractured zone (Fig. 4b). Therefore, the measured maximum height of fractured water-conducting zone of the first mining face in the study area is 106 m. Based on the vertical distribution of aquifers in the study area and the measurement data of surrounding mines^23^, we could conclude that the direct water-bearing aquifers in the study area are sandstone aquifer in the roof of the 3^–1^ coal seam in the third member of the Yan’an Formation and sandstone aquifer in the roof of the 2^–2^ coal seam of the first member of the Zhiluo Formation.

#### Sedimentary characteristics control the lateral water-richness of aquifers

**Figure 5:**
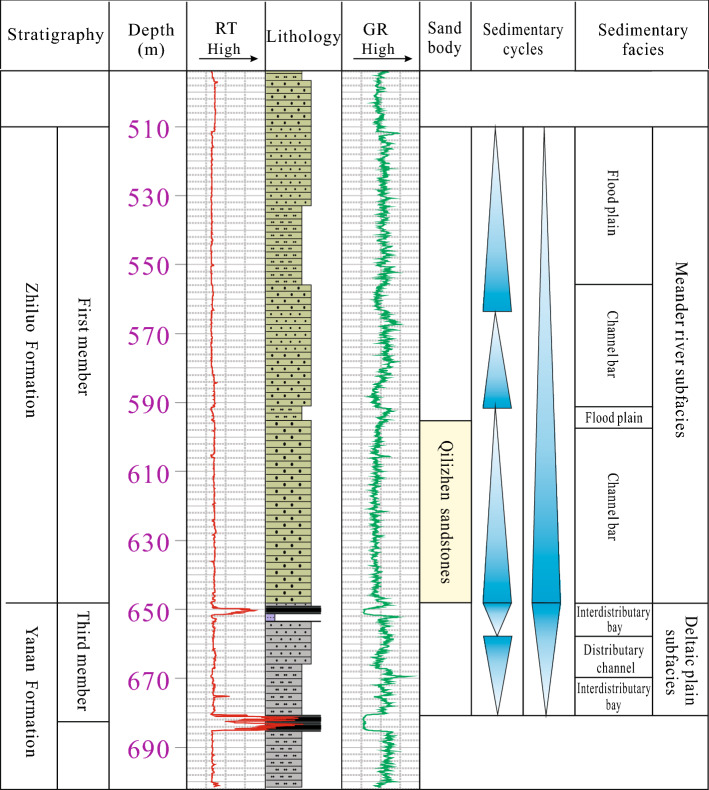
Sedimentary facies analysis of well H80.

**Figure 6:**
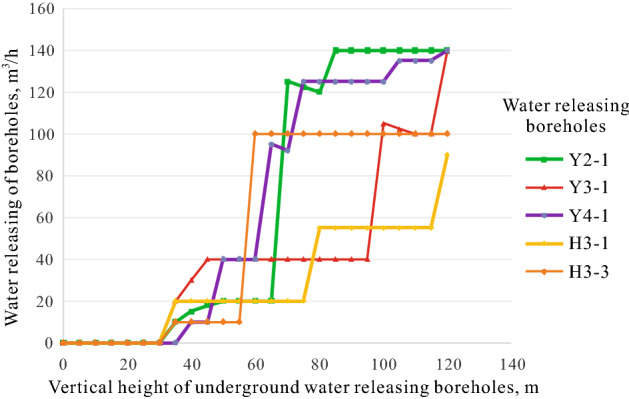
Water releasing results of underground boreholes in the first working face.

In study area, the water-bearing aquifers are divided into two mid-term cycles in the third member of Yan'an Formation and the first member of Zhiluo Formation according to the data of 104 boreholes, the logging facies markers, and regional sedimentary characteristics (Fig. 5). Rocks of the third member of the Yan'an Formation mainly consist of grayish-white medium- and fine-grained sandstone, which are sub-circular and moderately sorted with quartz and feldspar as the main mineral components. The subfacies of Lacustrine delta plain deposits have been developed, and are largely characterized by the asymmetric cycle of base-level decline. Distributary channel deposits act as a skeleton, and the rocks of the sand bodies are mainly composed of fine sandstone, generally showing a sorted and lenticular profile. These conditions are not conducive to the development of groundwater. According to the results of underground water releasing boreholes in the first working face of study area (Fig. 6), the 0–40 m vertical height of the boreholes is within the third member of the Yan'an Formation with low water release. This indicates the water-richness of the middle-fine grained sandstone aquifer in the third member of Yan'an Formation where the subfacies of delta plain are the main sedimentary environment is relatively weak.

Rocks in the first member of the Zhiluo Formation mainly consist of gray-green medium-coarse-grained sandstone with quartz and feldspar, and are medium-well sorted. Meandering river facies deposits have mainly been developed in this section, which shows an asymmetric cyclonic feature being dominated by the rise of the datum. The sandstone aquifer, which has a great impact on the mined coal seam, has been mainly developed in the subfacies of channel bar in the lower part of the first member of the Zhiluo Formation. This Formation has been continuously superimposed by multiple channel sand bodies with good connectivity, stable spatial distribution and sound space for groundwater storage. According to Fig. 6, the 40–120 m vertical height of the boreholes is within the first member of the Zhiluo Formation with water release increasing significantly, indicating that the medium-coarse-grained sandstone aquifer in the first member of the Zhiluo Formation where the subfacies of channel bar are the main sedimentary environment is relatively water-rich.

#### Sedimentary characteristics control the plane water-richness of aquifers

**Figure 7:**
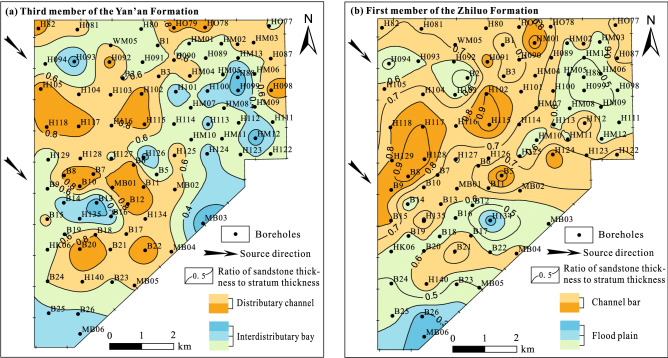
Distribution of sedimentary facies of the Yan’an and Zhiluo Formation.

We combined the ratio of sandstone thickness to stratum thickness in the mid-term datum cycle with the characteristics of the sedimentary microfacies distribution obtained through stratum thickness, sandstone thickness and logging markers, and recovered the sedimentary facies distribution of the third member of the Yan’an Formation and the first member of the Zhiluo Formation in the study area (Fig. 7). The third member of the Yan'an Formation in the study area was formed during the period of evolution from lacustrine delta plain subfacies to fluvial facies, when the ratio of accommodating space to sediment supply was small, and the distributary channel microfacies and interdistributary bay microfacies have been mainly developed in delta plain subfacies.

The distributary channel microfacies provide a better space for the occurrence of groundwater that is distributed from northwest to southeast part of the study area. The first member of the Zhiluo Formation is mainly composed of channel bar microfacies and floodplain microfacies. The channel bar microfacies are mainly distributed in the central and southern part of the study area. This period is of less accommodating space with stronger hydrodynamic conditions, as well as sheet-like shaped multi-phase channel sand bodies of continuous superposition, which is the most important storage space for groundwater in the water-bearing aquifers of the study area.

### Evaluation of the water-richness zoning based on sedimentary characteristics

#### Establishment of thematic maps of the main controlling factors

**Figure 8:**
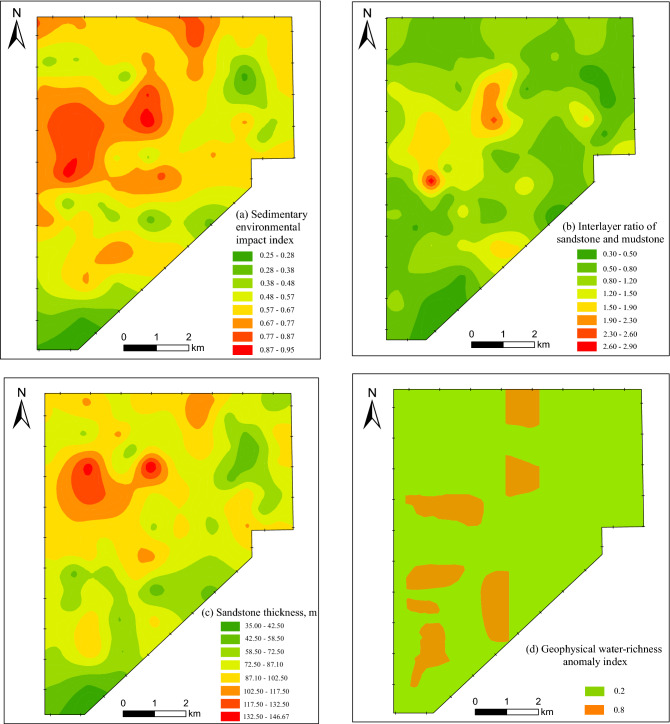
Thematic maps of the main controlling factors.

To analyze the sedimentary characteristics of roof water-bearing aquifers in the coal seam of the study area, four indicators are selected as the main controlling factors for water-richness evaluation, i.e., sedimentary environment impact index (B_1_), interlayer ratio of sandstone and mudstone (B_2_), sandstone thickness (B_3_), and geophysical water-richness anomaly index (B_4_). Among them, the value of sedimentary environmental impact index is determined based on the average value of underground water releasing from the third member of the Yan'an Formation and the first member of the Zhiluo Formation, which has been obtained by superimposing the sedimentary facies distribution of the two members with the weight of 1:4. Furthermore, through the full mining data of the boreholes in the study area, we have established the information thematic maps of each controlling factor, as shown in Fig. 8.

#### Model and results of the water-richness evaluation

**Figure 9:**
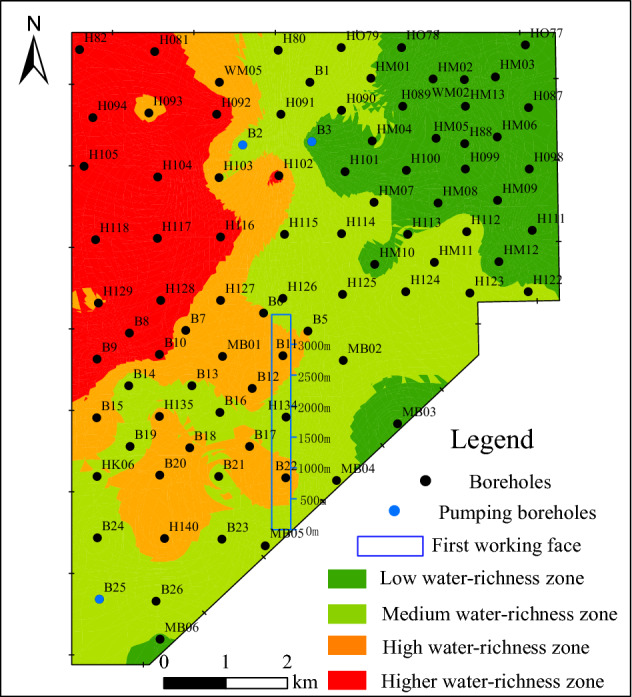
Zoning map of water-richness for roof water-bearing aquifers.

It is one of the key steps to determine the weights of the evaluation indicators in the water-richness evaluation of roof water-bearing aquifers. Firstly, we obtain the weighing results of each indicator through AHP, which denotes as (B_1_, B_2_, B_3_, B_4_) = (0.375, 0.125, 0.250, 0.125). Secondly, we use the GIS spatial information fusion function to superpose the four thematic maps of the main controlling factors, and obtain a comprehensive index that reflect the water-richness of the roof aquifers. Finally, we adopted the GIS natural classification method to process the zone thresholds of the comprehensive index, and obtain the evaluation results of water-richness zoning of the water-bearing aquifers in the roof of the 3^–1^ coal seam in the study area, as shown in Fig. 9.

#### Analysis and verification of evaluation results

It can be seen from Fig. 9 that the water-richness distribution of the water-bearing aquifers in the roof of the 3^–1^ coal seam is directional. Areas with high water-richness are mainly located in the west and northwest of the study area. These areas are multi-phase palaeochannels areas, and are dominated by the development of thicker medium-coarse-grained sandstone, which have been subjected to relatively strong weathering due to the later tectonic movement. Therefore, the sandstone strata in these areas have good pore structure and connectivity with high water-rich. In contrast, the areas with low water-richness are mainly located in the east and south of the study area, which are mainly floodplain deposits or interdistributary bay deposits consisting of gray or purple sandy mudstone or siltstone, and are interspersed with small channel sand bodies. The stratas in these areas are weakly weathered, and have poor pore structure, connectivity, and low water-rich.

Within the study area, there are only three pumping boreholes (B_2_, B_3_, B_25_) tapped water-bearing aquifers in the roof of the 3^–1^ coal seam with the unit water inflow of 0.16 L/(s m), 0.0567 L/(s m) and 0.106 L/(s m), respectively. According to the "Rules of Coal Mine Prevention and Control", the peripheral areas of B_2_ and B_25_ pumping boreholes are medium water-richness zones, and the peripheral area of B3 pumping borehole is low water-richness zone. The evaluation results are well consistent with the actual pumping test results.

**Figure 10:**
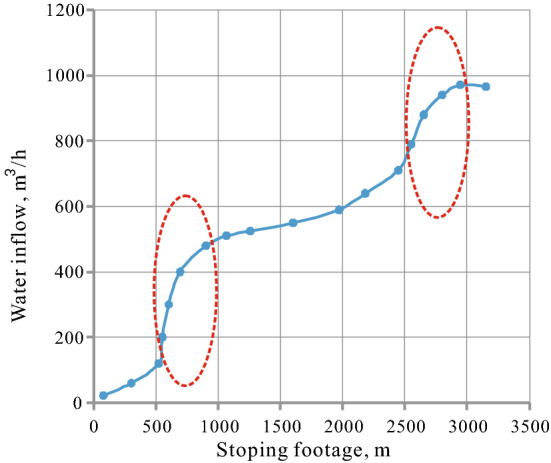
Correlation of between stoping footage and water inflow in the first working face.

According to Fig. 10, the water inflow of the first working face increases gradually with the increase of stoping footage. When the working face is mined to 500–1000 m and 2500–3000 m, the slope of the curve increases significantly reflecting that the water inflow of the working face increases significantly. This indicates that the aquifers in these two areas are high water-rich. It could be seen from Fig. 8 that the evaluation results based on sedimentary characteristics are consistent with the actual water inflow. Therefore, the application of this evaluation method in the study area is feasible and effective under the premise that the boreholes of the pumping test are few and cannot fully reflect the water-richness distribution of regional aquifers.

## Conclusion

This paper proposes a sediment control method for water-richness law and zoning in the roof aquifers of deep Jurassic coals. The method can be used to analyze the pattern of the plane and lateral spatial distribution of the water-bearing aquifers and the water-richness distribution from genesis, and to innovatively construct and define the evaluation indicator of sedimentary environmental impact index to quantitatively depict the impact of the distribution of sedimentary facies on the water-richness distribution of water-bearing aquifers. Sediment control method is adopted to construct a comprehensive evaluation system and establish a water-richness zoning map, which improves the accuracy of the water-richness evaluation, and provide a reference for the analysis and evaluation of water-richness of roof aquifers in mining areas without hydrogeological tests.

This method has been applied to the Muduchaideng Coal Mine in the Huji'erte mining area. The measured maximum height of fractured water-conducting zone of the first mining face in the study area is 106 m. The water-bearing aquifers in the study area are sandstone aquifer in the roof of the 3^–1^ coal seam in the third member of the Yan’an Formation and sandstone aquifer in the roof of the 2^–2^ coal seam of the first member of the Zhiluo Formation. The sandstone aquifer in the first member of the Zhiluo Formation is mainly composed of channel bar microfacies with good spatial continuity of the sand body, and is relatively water enriched. A water-richness zoning model is established based on sedimentary characteristics, and the high water-rich zones are mainly located in the west and northwest of the study area, which are multi-phase palaeochannels areas. The zoning evaluation results of water-richness have been verified with limited pumping boreholes and mine water inflow, and are well consistent with the actual situation (Supplementary information).

## Supplementary Information

Supplementary Information.

## Data Availability

The datasets used and/or analysed during the current study available from the corresponding author on reasonable request.
